# Rapid resolution of an acute subdural hematoma by increasing the shunt valve pressure in a 63-year-old man with normal-pressure hydrocephalus with a ventriculoperitoneal shunt: a case report and literature review

**DOI:** 10.1186/1752-1947-6-393

**Published:** 2012-11-22

**Authors:** Jackson Hayes, Marie Roguski, Ron I Riesenburger

**Affiliations:** 1 Department of Neurosurgery, Proger 7, Tufts Medical Center, 800 Washington St, Boston, MA 02111, USA

**Keywords:** Normal-pressure hydrocephalus, Ventriculoperitoneal shunt, Acute subdural hematoma, Programmable shunt valve

## Abstract

**Introduction:**

Symptomatic subdural hematoma development is a constant concern for patients who have undergone cerebrospinal fluid shunting procedures to relieve symptoms related to normal-pressure hydrocephalus. Acute subdural hematomas are of particular concern in these patients as even minor head trauma may result in subdural hematoma formation. The presence of a ventricular shunt facilitates further expansion of the subdural hematoma and often necessitates surgical treatment, including subdural hematoma evacuation and shunt ligation.

**Case presentation:**

We present the case of a 63-year-old North American Caucasian man with normal-pressure hydrocephalus with an adjustable valve ventriculoperitoneal shunt who developed an acute subdural hematoma after sustaining head trauma. Conservative treatment was favored over operative evacuation because our patient was neurologically intact, but simple observation was considered to be too high risk in the setting of a low-pressure ventriculoperitoneal shunt. Thus, the valve setting on the ventriculoperitoneal shunt was increased to its maximum pressure setting in order to reduce flow through the shunt and to mildly increase intracranial pressure in an attempt to tamponade any active bleeding and limit hematoma expansion. A repeat computed tomography scan of the head six days after the valve adjustment revealed complete resolution of the acute subdural hematoma. At this time, the valve pressure was reduced to its original setting to treat symptoms of normal-pressure hydrocephalus.

**Conclusions:**

Programmable shunt valves afford the option for non-operative management of acute subdural hematoma in patients with ventricular shunts for normal-pressure hydrocephalus. As illustrated in this case report, increasing the shunt valve pressure may result in rapid resolution of the acute subdural hematoma in some patients.

## Introduction

Normal-pressure hydrocephalus (NPH) usually presents in older patients. Patients with NPH frequently present with ventricular dilation, dementia, magnetic gait and urinary incontinence. In 1965, Adams *et al*. postulated a gradual blockage of cerebrospinal fluid (CSF) drainage as the underlying cause [1]; however, in most patients, NPH is idiopathic and the etiology remains largely unknown. Management of patients with NPH involves placement of a ventriculoperitoneal shunt to aid CSF drainage. The placement of adjustable shunt valves enables easy, non-invasive adjustments in the amount of CSF drainage in order to maximize symptom relief, minimize over-drainage, thus reducing the need for repeated surgical interventions to manage shunt pressure with fixed pressure valves [2-8].

A major risk involving both fixed and adjustable ventricular shunts is a predisposition to subdural hematoma (SDH) development. These patients are susceptible to SDH formation due to reduced intracranial pressure (ICP) caused by over-drainage of CSF, acute trauma to the head, or both [2,5,6,8-11]. Samuelson *et al*. reported that, following successful ventricular shunt placement and relief of NPH symptoms, five of 24 patients were readmitted for SDH between one and 11 months post-operatively, with one of the five cases reporting a history of trauma. Additionally, patients with NPH were found to be particularly susceptible to SDH formation following ventricular shunt placement, in contrast to a comparatively lower incidence of SDH development after treatment for high-pressure hydrocephalus [7].

Treatment of acute SDH in patients with NPH is often difficult. Thus, in addition to evacuation of the SDH through burr holes or through a craniotomy, shunt ligature is sometimes needed to prevent reaccumulation or expansion of SDH. We report the case of a patient with NPH who sustained head trauma and developed an acute SDH. Our patient was successfully managed non-operatively to achieve rapid resolution of the acute SDH.

## Case presentation

A 63-year-old North American Caucasian man was admitted to our hospital after falling down four steps and sustaining head trauma. At presentation, he stated he had a headache, but denied visual changes, numbness or weakness. He was neurologically intact; notably, pronator drift was absent. His medical history was significant for NPH for which a programmable Medtronic Strata® ventriculoperitoneal (VP) shunt was placed three years prior to this event.

**Figure 1:**
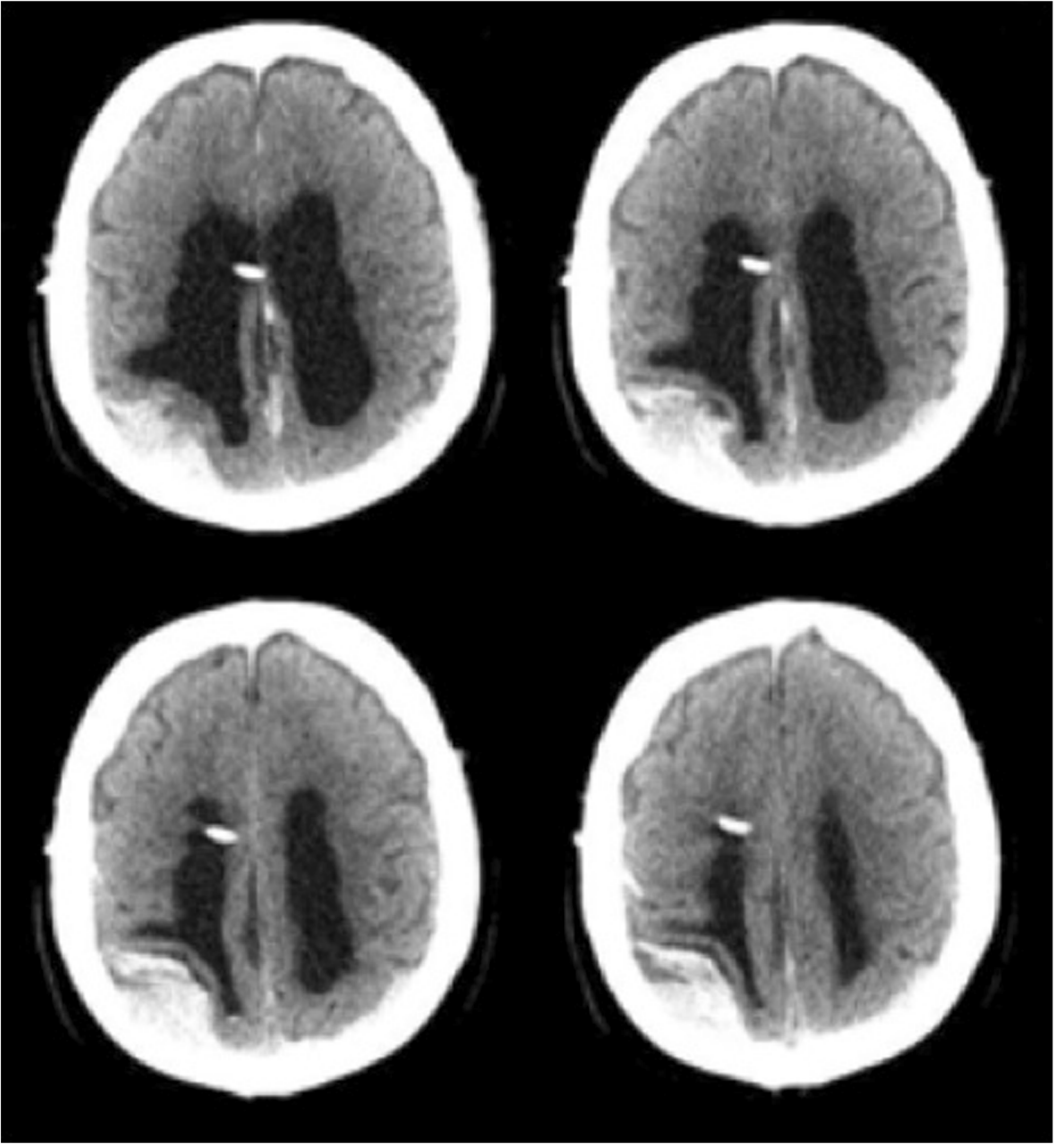
**Pre-valve adjustment computed tomography scan.** The pre-valve adjustment computed tomography scan showing a high-density subdural hematoma in the right convexity and a mixed density subdural hematoma in the left convexity.

A computed tomography (CT) examination showed the ventricular catheter and an acute right posterior convexity subdural hematoma (Figure 1). The SDH overlying the right convexity measured 3cm in the greatest transverse diameter, causing mass effect on the ipsilateral brain parenchyma and posterior horn of the lateral ventricle.

Our patient was admitted to the intensive care unit for close neurological monitoring. Our patient was neurologically intact, and, thus, conservative management was favored over operative treatment. However, given the propensity for expansion of acute subdural hematomas in the presence of low-pressure ventriculoperitoneal shunts and given the moderate size of our patient’s subdural hematoma, simple observation was thought to be high risk. In addition, our patient reported dramatic improvement of his NPH symptoms after shunting, and, thus, simple shunt closure via ligature was not optimal. The authors chose to treat our patient’s acute subdural hematoma by utilizing a technique that has been used in the treatment of subdural hygromas and chronic subdural hematomas. This technique, wherein the programmable valve setting is changed to reduce CSF drainage, allowed conservative management and observation of our patient without operative intervention.

**Figure 2:**
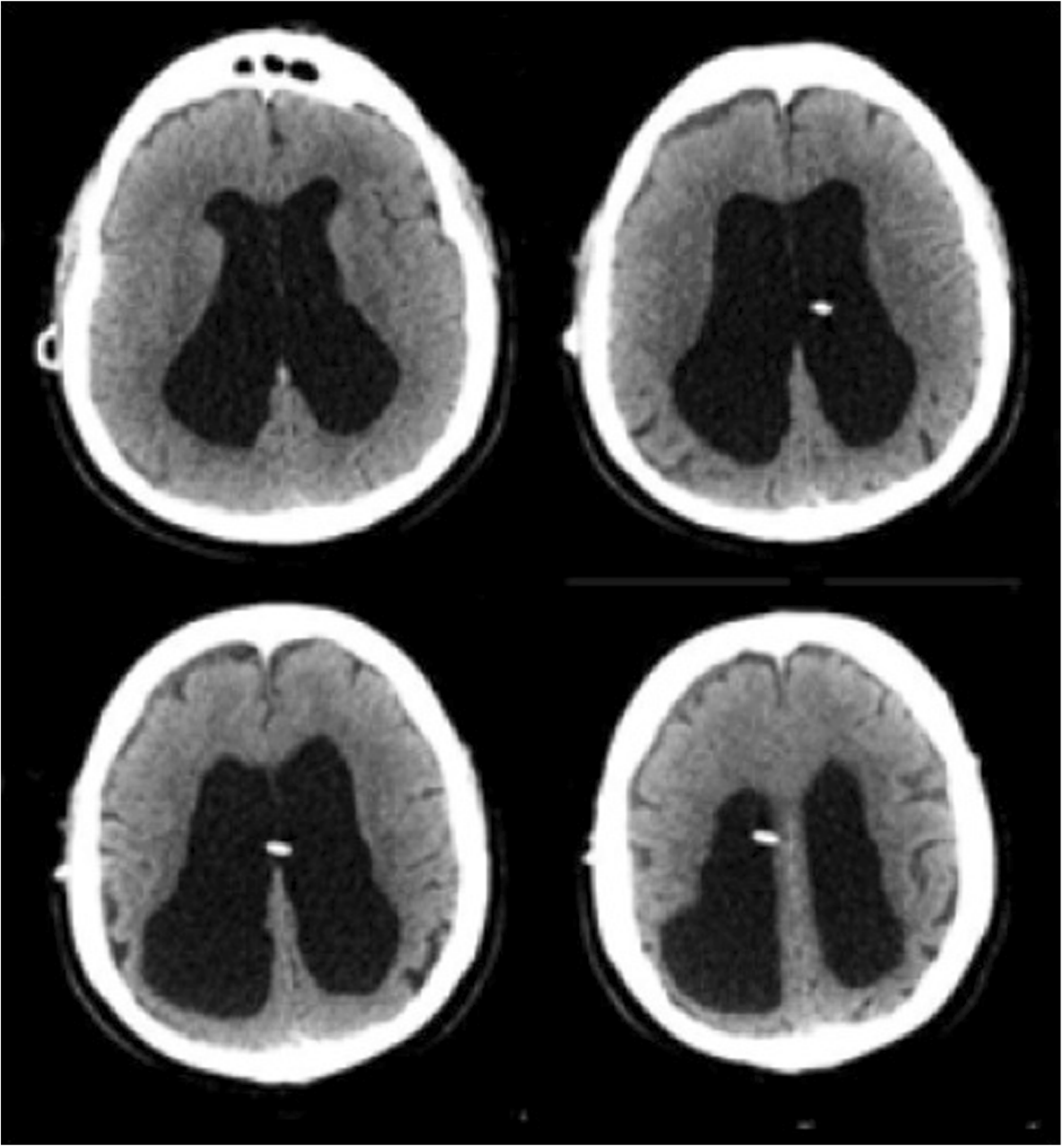
**Computed tomography scan conducted six days after valve adjustment.** The computed tomography scan obtained six days after valve adjustment shows ventricular dilation, residual trace bifrontal extra-axial hypodense collections, and encephalomalacia in the right posterior parietal lobe.

**Figure 3:**
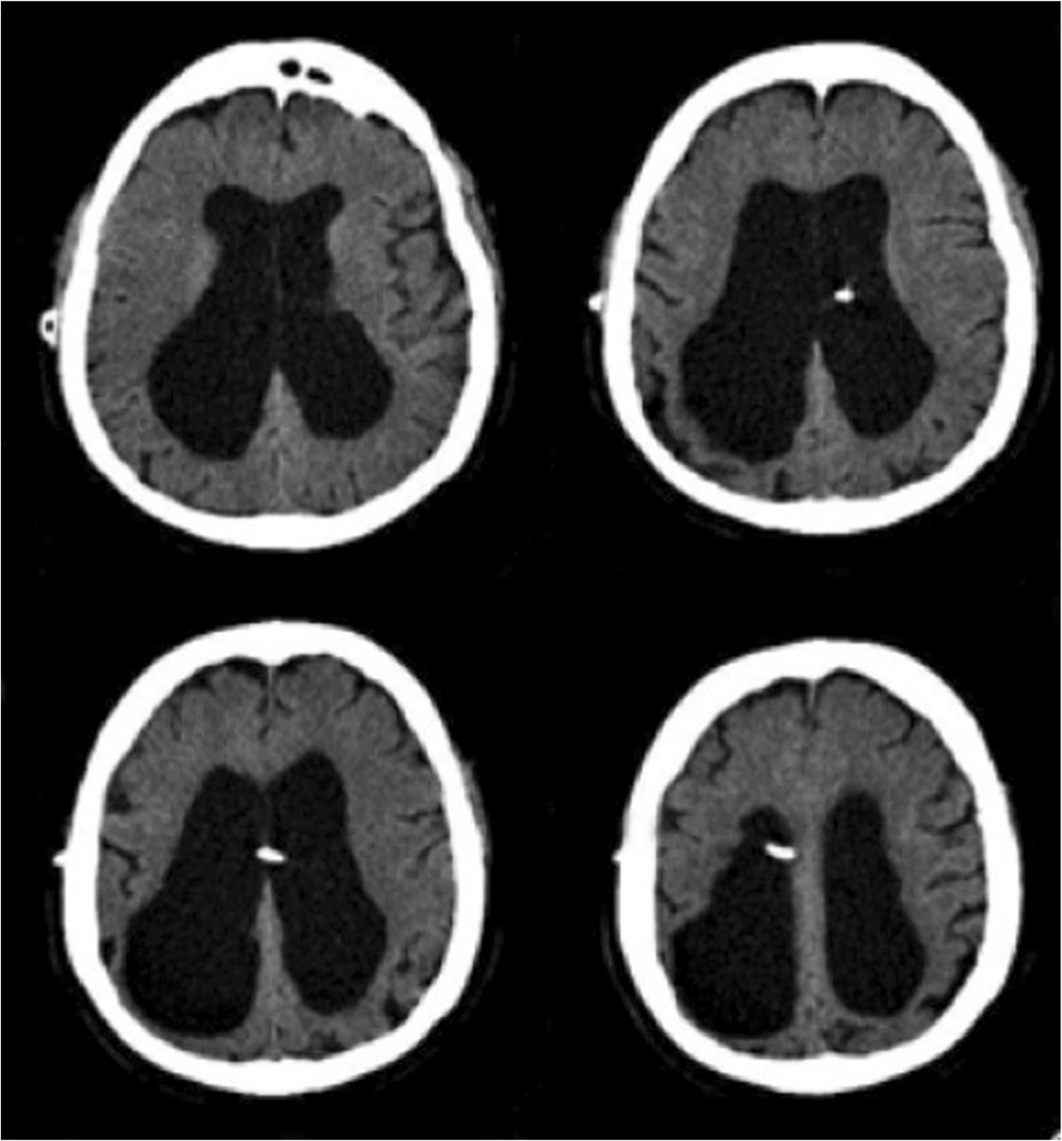
**Computed tomography scan one year after subdural hematoma.** The computed tomography scan performed one year after subdural hematoma shows resolution of the subdural hematoma and dilated ventricles.

The programmable valve was adjusted transcutaneously from 1.0 to the maximum setting of 2.5, thereby reducing CSF drainage. A repeat head CT obtained the following day revealed no significant change in the size of the subdural hematoma. He remained clinically unchanged and neurologically intact. He was then discharged from the hospital with a plan for close follow-up. Six days later, our patient was admitted with worsening symptoms of NPH including gait ataxia and urinary incontinence. CT examination showed complete resolution of the acute SDH and dilated ventricles consistent with our patient’s known history of NPH (Figure 2). The valve setting was reduced from 2.5 to 0.5 in order to promote greater CSF drainage. This alleviated the NPH symptoms. Our patient remains well one year after sustaining the traumatic subdural hematoma (Figure 3).

## Discussion

Programmable shunt valves have become a valuable tool in hydrocephalus management. The option to non-invasively change valve settings has been reported as advantageous over conventional fixed valves during management of NPH, hydrocephalus, chronic SDH, SDH and ventricular collapse due to over-drainage, and preventing SDH recurrence following evacuation [2,4,6,12-14]. The Dutch Normal-Pressure Hydrocephalus study showed that 71 percent of patients with fixed low-pressure valves developed subdural effusions compared to 34 percent for patients with fixed medium-pressure valves [3]. Thus, the development of subdural effusions seems to be directly related to the amount of CSF drainage.

Adjustable valves not only provide the ability to non-surgically increase valve settings to prevent subdural effusions, but also provide the added benefit of high-pressure drainage when needed to aid in the treatment of subdural effusions. Carmel *et al*. reported the use of shunt ligation and high-pressure drainage to treat a subdural fluid collection in a patient with NPH. Initially, shunt ligation was performed on a patient in an attempt to obliterate the subdural fluid collection, which unfortunately resulted in rapid deterioration of the patient. Due to worsening of patient’s clinical condition, the ligature was removed and the adjustable valve turned to a high-pressure setting. The adjustable valve provided the safety of high-pressure drainage, and the patient returned to baseline over five days [4].

Sindou *et al*. also reported on the treatment options offered by adjustable valves; in a series of 75 patients, 27 patients required non-operative valve adjustment to address hemispheric detachment, intracranial hypotension and absence of clinical improvement [13]. The benefit of adjustable shunts are further reinforced in a report by Zemack *et al*., which concluded that non-invasive shunt adjustment improved outcomes for patients with NPH after reporting a five-year adjustable shunt survival rate of 80.2 percent, with good to excellent outcomes observed in 78.1 percent of people with idiopathic NPH [14].

The literature has demonstrated benefit in lowering the incidence and treating chronic subdural hematomas in patients with NPH with adjustable shunt valve placement. However, there is very scant literature describing acute SDH management in shunted patients with NPH. This is surprising as the development of acute SDH after shunting for NPH is a serious concern. Even minor head trauma may lead to an acute SDH, necessitating surgical intervention [9,10]. Increased susceptibility to SDH can be attributed to the shunt decreasing intracranial pressure by decreasing ventricular volume, causing the vessels bridging the subdural space to become stretched and making them vulnerable to sheering forces and bleeding upon minor trauma [8-10]. Once bleeding begins, the ventricular shunt facilitates hematoma development by preventing the brain from tamponading further bleeding [15]. Furthermore, SDH development have been attributed to over-drainage of CSF alone, highlighting the strain experienced by subdural bridging vessels in patients with ventriculoperitoneal shunts [2,5,7,8,11,12].

Hoya *et al*. reported one case of non-operative acute SDH management in a patient with normal-pressure hydrocephalus and an adjustable valve [6]. They adjusted the valve to the highest setting in order to decrease CSF drainage. This patient unfortunately died from complications that appeared to be unrelated to the subdural hematoma. Hoya’s results are in contrast with the case that we present. Our patient was readmitted after six days following issues of NPH symptoms, including gait ataxia, generalized weakness and urinary incontinence. A CT of the head obtained upon readmission revealed complete resolution of the acute SDH and not just a reduction in volume. The valve pressure was then reduced to treat the NPH symptoms, and our patient’s neurological condition improved upon discharge. To the best of our knowledge, this is the first case detailing the results of non-operative shunt valve management of an acute SDH.

## Conclusions

Adjustable shunt valve management alone may serve as a non-invasive alternative to shunt ligature or subdural evacuation in shunted patients with NPH with an acute SDH at their neurological baseline. Once the SDH has resolved, pressure valve settings can again be lowered to optimize relief of NPH.

## Consent

Written informed consent was obtained from the patient for publication of this case report and any accompanying images. A copy of the written consent is available for review by the Editor-in-Chief of this journal.

## Competing interests

The authors declare that they have no competing interests.

## Authors’ contributions

JH analyzed and interpreted our patient’s chart, reviewed the literature, and prepared the manuscript. MR and RR made revisions and made major contributions in writing the manuscript. All authors read and approved the final manuscript.

## References

[B1] Adams RD Fisher CM Hakim S Ojemann RG Sweet WH Symptomatic occult hydrocephalus with “normal” cerebrospinal-fluid pressure. A treatable syndrome N Engl J Med 1965 273 117 126 10.1056/NEJM196507152730301 14303656

[B2] Black PML Hakim R Bailey NO The use of the Codman-Medos programmable Hakim valve in the management of patients with hydrocephalus: Illustrative cases Neurosurgery 1994 34 1110 1113 10.1227/00006123-199406000-00040 8084404

[B3] Boon AJ Tans JT Delwel EJ Egeler-Peerdeman SM Hanlo PW Wurzer HA Avezaat CJ de Jong DA Gooskens RH Hermans J Dutch normal-pressure hydrocephalus study: randomized comparison of low- and medium-pressure shunts J Neurosurg 1994 88 490 495 10.3171/jns.1998.88.3.0490 9488303

[B4] Carmel PW Albright AL Adelson PD Canady A Black P Boydston W Kneirim D Kaufman B Walker M Luciano M Pollack IF Manwaring K Heilbrun MP Abbott IR Rekate H Incidence and management of subdural hematoma/hygroma with variable and fixed pressure differential valves: a randomized, controlled study of programmable compared with conventional valves Neurosurg Focus 1999 7 Article 7 10.3171/foc.1999.7.4.2 16918220

[B5] Chrissicopoulos C Mourgela S Kirgiannis K Sakellaropoulos A Ampertos N Petritsis K Spanos A What is the appropriate shunt system for normal-pressure hydrocephalus? Acta Neurochir Suppl 2012 113 119 121 10.1007/978-3-7091-0923-6_24 22116436

[B6] Hoya K Tanaka Y Uchida T Takano I Nagaishi M Kowata K Hyodo A Treatment of traumatic acute subdural hematoma in adult hydrocephalus patients with cerebrospinal fluid shunt Clin Neurol Neurosurg 2011 114 211 216 22030155 10.1016/j.clineuro.2011.10.002

[B7] Samuelson S Long D Chou S Subdural hematoma as a complication of shunting procedures for normal pressure hydrocephalus J Neurosurg 1972 37 548 551 10.3171/jns.1972.37.5.0548 5076372

[B8] Sternbach GL Subdural hematoma in a shunted patient J Emerg Med 2005 29 483 484 10.1016/j.jemermed.2005.05.008 16243213

[B9] Aoki N Mizutani H Acute subdural hematoma due to minor head trauma in patients with lumboperitoneal shunt Surg Neurol 1988 29 22 26 10.1016/0090-3019(88)90118-8 3336835

[B10] Kamiryo T Hamada J Fuwa I Ushio Y Acute subdural hematoma after lumboperitoneal shunt placement in patients with normal pressure hydrocephalus Neurol Med Chir (Tokyo) 2003 43 197 200 10.2176/nmc.43.197 12760499

[B11] McCullough DC Fox JL Negative intercranial pressure hydrocephalus in adults with shunts and its relationship to the production of subdural hematoma J Neurosurgery 1974 40 372 375 10.3171/jns.1974.40.3.0372 4813717

[B12] Kamano S Nakano Y Imanashi T Hattori M Management with a programmable pressure valve of subdural hematomas caused by a ventriculoperitoneal shunt: case report Surg Neurol 1991 35 381 383 10.1016/0090-3019(91)90050-J 2028387

[B13] Sindou M Guyotat-Pelissou I Chidiac A Goutelle A Transcutaneous pressure adjustable valve for the treatment of hydrocephalus and arachnoid cysts in adults Acta Neurochir (Wien) 1993 121 135 139 10.1007/BF01809264 8512009

[B14] Zemack G Romner B Adjustable valves in normal-pressure hydrocephalus: a retroperspective study of 218 patients Neurosurgery 2002 51 1392 1401 12445344

[B15] Aoki N Lumboperitoneal shunt: clinical applications, complications, and comparison with ventriculoperitoneal shunt Neurosurgery 1990 26 998 1004 10.1227/00006123-199006000-00013 2362678

